# Multi-scale structural characterization of ceramic-based photonic glasses for structural colors

**DOI:** 10.1186/s11671-024-04057-x

**Published:** 2024-07-08

**Authors:** Yen Häntsch, Ana Diaz, Mirko Holler, Tobias Krekeler, Martin Ritter, Sabine Rosenfeldt, Markus Retsch, Kaline P. Furlan

**Affiliations:** 1 Institute of Advanced Ceramics, Hamburg University of Technology, Denickestraße 15, 21073 Hamburg, Germany; 2 Paul Scherrer Institute, Forschungsstrasse 111, 5232 Villigen, PSI Switzerland; 3 Electron Microscopy Unit, Hamburg University of Technology, Eißendorfer Straße 42, 21073 Hamburg, Germany; 4 Department of Chemistry, University of Bayreuth, Universitaetsstr. 30, 95447 Bayreuth, Germany; 5 Institute of Advanced Ceramics, Integrated Materials Systems Group, Hamburg University of Technology, Denickestraße 15, 21073 Hamburg, Germany

**Keywords:** Structural colors, Ptychography X-ray computed tomography, 3D image analysis, Ceramics, Photonic materials

## Abstract

Structural colors arise from selective light interaction with (nano)structures, which give them advantages over pigmented colors such as resistance to fading and possibility to be fabricated out of traditional low-cost and non-toxic materials. Since the color arises from the photonic (nano)structures, different structural features can impact their photonic response and thus, their color. Therefore, the detailed characterization of their structural features is crucial for further improvement of structural colors. In this work, we present a detailed multi-scale structural characterization of ceramic-based photonic glasses by using a combination of high-resolution ptychographic X-ray computed tomography and small angle X-ray scattering. Our results uncover the structure-processing-properties’ relationships of such nanoparticles-based photonic glasses and point out to the need of a review of the structural features used in simulation models concomitantly with the need for further investigations by experimentalists, where we point out exactly which structural features need to be improved.

**Supplementary Information:**

The online version contains supplementary material available at 10.1186/s11671-024-04057-x.

## Introduction

**Fig. 1:**
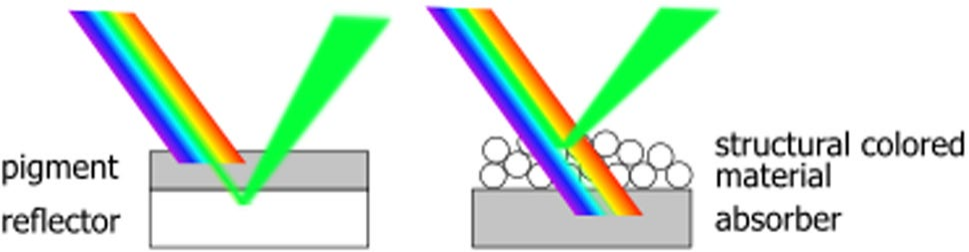
Schematic drawing of the comparison between the coloration mechanism of pigments and structural colors. In pigments, like chlorophyll, the light is selectively absorbed and the remaining light is reflected back giving rise to coloration. Structural colors as observed in opal gemstones are a result of selective reflection of light by the nanostructure while the remaining wavelengths are transmitted through it (and potentially absorbed by an underlaying absorber layer)

A large variety of structures and films have their coloration defined by the presence of pigments and dyes, for which the color arises due to selective light absorption [1]. Leaves, for example, are mostly green because chlorophyll absorbs light at 400–500 nm and 600–700 nm ranges, i.e. blue and red light are absorbed [2], while green light is reflected and perceived as color. Meanwhile, structural coloration arises from selective light interaction with complex nanostructures [3–5]. In such case, green color will be perceived when the photonic structure reflects green light while light related to other colors remains “trapped”, passes through the material and ends up being absorbed by an underlying broadband absorber layer (Fig. 1).

While the schematic drawing of Fig. 1 and associated basic explanation can facilitate the understanding of the differences between pigmented and structural color, in reality, the structural coloration arises from fundamental optical processes involving reflection, refraction, diffraction, interference and scattering of light [6]. To achieve selective light reflection in the range of visible light, structural features on the dimension comparable to the wavelength of visible light are required [7]. This gives structural colors its big advantage over pigmented colors: they will be stable as long as such structural features remain unaltered [8]. Moreover, since they are based on the interaction of light with its nanostructures, they can be fabricated out of traditionally “white” or even “transparent” materials, such as titania [3] and silica [9]. Meanwhile, pigmented colors are prone to fading due to pigment degradation [6, 10] and have to be fabricated out of chemical compounds that specifically absorb photons at a certain wavelength range, being sometimes toxic such as cadmium selenide used for red color.

Three-dimensional (3D) structural colors found in nature present a vast variety of complex nanostructures [11], where the arrangement of the structural units responsible for the interaction with light can be divided into ordered or disordered. In general, periodically-ordered structures are called 3D photonic crystals (PhCs), whereas disordered structures have received different names such as amorphous PhCs [12, 13], colloidal amorphous arrays, [9], glassy photonic films [14], and photonic (colloidal) glasses (PhGs) [15, 16]. In this work we use the PhG nomenclature. Aside from the structural ordering, the biggest difference between PhCs and PhGs is that ordered structures will deliver a color which is iridescent, i.e. the color appearance changes depending on the angle of observation, whilst PhGs provide non-iridescent structural colors, i.e. the color perception is the same from any observation angle which can be seen as an advantage [6].

In 3D PhGs where the structure is based on nano to submicron spherical building blocks, hereby called motif, a subdivision can be made in between direct and inverted structures. While direct PhGs consist of an arrangement of solid spherical particles [9, 11, 15, 17–21], the broad classification of inverse PhGs include any other structure generated by the negative of a direct PhG [22]. This can be performed by using infiltration methods such as sol–gel, chemical vapor deposition and atomic layer deposition [3], but also via colloidal methods using co-deposition [23] or heterocoagulation [8, 24]. Depending on the fabrication process, the inverse PhGs might be further subdivided into surface templated [25] or hollow-sphere type [8]. The selected fabrication process(es) will define not only the PhG composition, but also the structural features which directly influence its photonic properties and thus, their final color. Among the structural features that can impact the photonic response are the packing density, the degree of disorder, the building block size and the effective refractive index of the structure [26–28]. The latter is dependent not only on the PhG material and the environment material (usually air), but specially for inverse structures also the shell size [29] and density [26] with an intricate relation between motif size and spatial arrangement [4].

Therefore, a proper characterization of the structural features of hollow-sphere PhGs, such as shell thickness, macropore size and packing density are crucial for further development of structural colors. However, the characterization of these complex multiscale structures is quite challenging. Being a fast and facile tool, scanning electron microscopy (SEM) is still the main technique used for characterization of PhGs’ structural features. However, such technique presents several limitations, the biggest being that only qualitative 2D information is obtained. For hollow-sphere PhGs, where the shell thicknesses are in the order of 10 to 50 nm, lack of resolution to resolve the finest features can also be an issue. At such, transmission electron microscopy (TEM) imaging might be an alternative for analysis of the shells, however missing information on 3D structural features such as packing fraction. Moreover, with both TEM and SEM it is only possible to visualize one “layer” at each analysis. Alternatively, X-ray computed tomography and especially high-resolution synchrotron-based ptychographic X-ray computed tomography (PXCT) presents a great opportunity to study the structural morphology of porous materials [30, 31], including multi-scale PhGs. The biggest advantage of PXCT over conventional SEM or TEM is the possibility to visualize and analyze many “layers” (area projections) for each sample, thus providing the possibility to identify 3D features that span over different layers.

In this work, we present a detailed multi-scale structural characterization of ceramic-based PhGs made out of Yttria-stabilized zirconia (YSZ) nanoparticles by using a combination of high-resolution PXCT and small angle X-ray scattering (SAXS). While PXCT provided us valuable information regarding several 3D structural features of the PhGs, SAXS delivered us information regarding the nanoparticles’ shell formation when in suspension. The obtained results are correlated with the optical properties of YSZ-PhGs fabricated with different parameters, where intricate relations between the fabrication parameters and the final properties were drawn. Our results uncover the reasons for reduced color saturation and point out to the potential need of a review of the structural features used during simulations of PhGs structures. They also provide valuable information for experimentalists to improve the heterocoagulation fabrication process of hollow-sphere PhGs.

## Methods

**Fig. 2:**
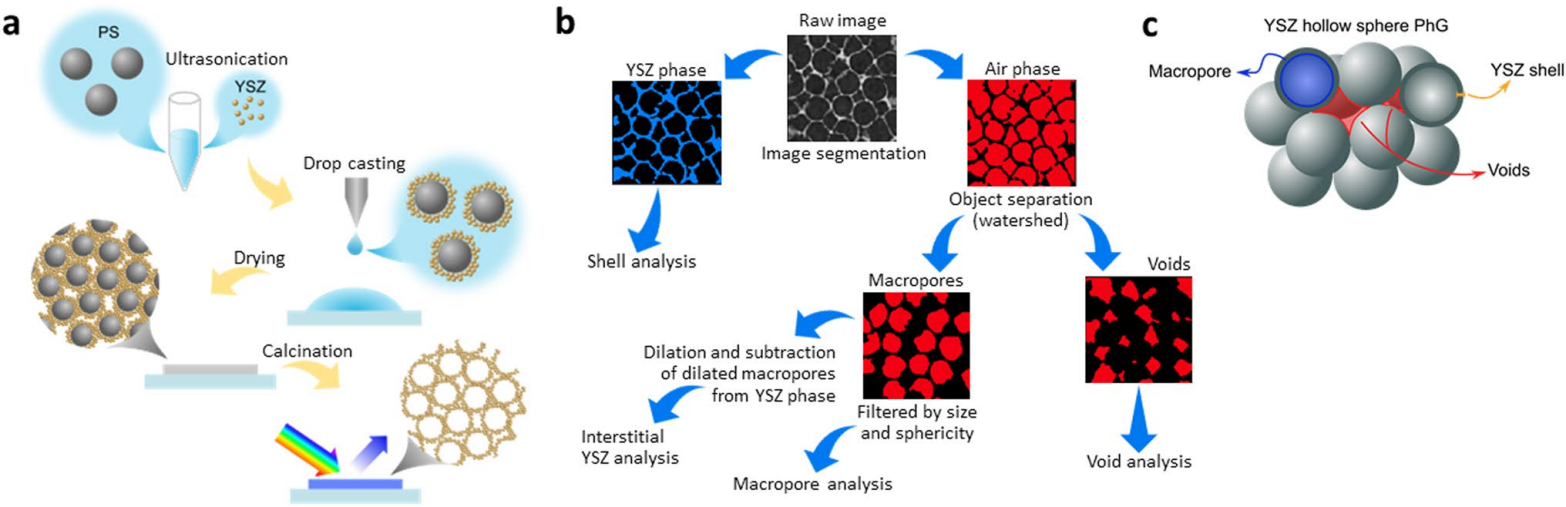
Schematic drawing of the **a** fabrication procedure of hollow-sphere YSZ PhGs and **b** image processing procedure for samples investigated via PXCT. The raw image from the sample reconstruction was segmented into YSZ phase and air phase with further classification into voids and macropores. Quantitative and qualitative analysis of the 3D features shown in **c** was carried out based on the classified objects. Three levels of characteristic length scales determine the mesostructure of the hollow sphere ensemble: templated macropores, aggregation dependent voids, and YSZ shells

The fabrication of YSZ hollow-sphere PhGs was performed according to a heterocoagulation process route [4, 23], summarized in Fig. 2a. For such, polystyrene (PS) monodisperse particles with 295 nm diameter were acquired from Microparticles GmbH, while YSZ nanoparticles with diameter under 10 nm were prepared by hydrothermal synthesis following the protocol described by Guiot et al. [32]. For the nanoparticles’ synthesis, zirconyl nitrate hydrate (ZrO(NO_3_)_2_·H_2_O, 99%, Sigma-Aldrich) and yttrium nitrate hexahydrate (Y(NO_3_)_3_·6H_2_O, 99.5%, Sigma-Aldrich) were dissolved in 60 mL ultrapure water (resistivity of 18.2 Ω cm obtained by a Millipore Milli-Q system) to a resulting concentration of 0.1 M for Zr^4+^ ions and 0.05 M for Y^3+^ ions followed by the addition of acetylacetone reaching the concentration of 0.1 M. Then, aqueous ammonia solution (3 M) was added dropwise until the mixture reached pH 7. The mixture was heated up to 160 °C in an autoclave for 3 days, followed by dialysis with ultrapure water using a tubular cellulose membrane (25–30 Å pore size) for purification and centrifugation at 5000 × g for 1 h. After removal of the supernatant, the resulting product is sonicated (Ultrasonic processor UP100H, Hielscher Ultrasonics) for 16 h resulting in a clear and stable suspension.

For the heterocoagulation process, a 13.8 mg/mL suspension of polystyrene particles is mixed with a YSZ nanoparticles’ suspension with opposite particle charges in ultrapure water and further sonicated for 30 min to ensure complete homogenization. The concentration of PS spheres was kept fixed while the YSZ nanoparticle concentration in the mixture was either 5.5 or 13.8 mg/mL resulting in YSZ/PS ratios of 0.4 and 1.0 by weight, hereby referred to as 0.4-PhG and 1.0-PhG, respectively. The heterocoagulated suspension was then drop-casted inside polytetrafluoroethylene (PTFE) rings with an inner diameter of 2 cm placed on top of silicon wafers (as received, < 100 > , native oxide layer, Si-Mat Silicon Materials) and dried under ambient conditions. For removal of the PS template, the as-cast films were calcined in a muffle furnace under air atmosphere at 500 °C for 30 min at a heating rate of 1 °C/min. The cooling rate followed the natural cooling of the muffle furnace with exponential decay and can be approximated to 1 °C/min for temperatures above 250 °C.

Cylindrical pillars of each hollow-sphere PhG were prepared by focused ion beam milling (FIB) to a final diameter of 5.67 µm and 5.85 µm for the 0.4-PhG and the 1.0-PhG compositions, respectively (Figure S1). FIB was performed using a FIB-SEM FEI Helios G3 UC. The whole sample is made conductive by carbon deposition using a carbon thread evaporator (Bal Tec CED 030). The region of interest was protected from FIB-damage by electron- and ion-deposition of carbon. The tomography sample was prepared using a circular milling pattern with 9.3 nA beam current. Transfer of the pillar to the tomography pin was done at 0° sample tilt using an in-situ nanomanipulator and ion-deposition of platinum. The deposited platinum and remnants of the tungsten manipulator needle are removed from the top of the sample using a cleaning cross sectioning pattern with 0.43 nA beam current. Final polishing of the pillar is done with 80 pA beam current.

Fibbed pillars were mounted on the tip of OMNY pins [33], especially designed for the instrument used for the PXCT measurements, conducted at the cSAXS beamline of the Swiss Light Source (SLS) at the Paul-Scherrer-Institut (PSI) in Villingen, Switzerland using an optimized high-resolution PXCT instrument developed by Holler et al. [34], and a photon energy of 6.2 keV. The instrument features relative interferometry between the sample and the Fresnel zone plate, which defines the illumination onto the sample allowing their relative positioning with an accuracy better than 5 nm. This is necessary for performing high-resolution X-ray ptychography [35], in which the sample is scanned across the illumination in such a way that neighboring illuminated areas partially overlap. At each scanning position, coherent diffraction patterns are recorded on a 2D detector in the far field, and iterative phase retrieval algorithms are used to reconstruct 2D images which represent the projected refractive index of the sample with both amplitude and phase contrast. The setup up allows automated rotation of the samples in their axis from 0 to 180 °C, while projections are acquired. The reconstructed phase of all 2D projections are then reconstructed tomographically to render the 3D electron density distribution of the specimen. The final 3D resolution assessed via Fourier shell correlation [36] was 8.9 nm and 10.5 nm for 0.4-PhG and 1.0-PhG, respectively. Details about PXCT data acquisition and reconstruction can be found in the Supplementary Information.

The resulting tomographic slices were post-processed with ImageJ and analyzed with Avizo 9.5 (Thermo Fisher). For the quantitative analysis of the hollow-sphere structure, a systematic image processing flow was performed, which is summarized in Fig. 2c and b, respectively. Further details about each step can be found in the Supplementary Information.

Small-angle X-ray scattering (SAXS) investigation of heterocoagulated YSZ nanoparticles and PS template spheres suspended in water was performed with a lab-based SAXS double Ganesha AIR system (SAXSLAB/Xenocs) with a Cu rotating anode (MicroMax 007HF, Rigaku Corporation, Japan, wavelength of 1.54 Å). The suspensions were filled into glass capillaries with diameter of 1 mm (Hilgenberg, Germany) and a water-filled glass capillary was used for background correction. For data acquisition, a position-sensitive detector (PILATUS 300 K, Dectris) was used. The obtained two-dimensional scattering patterns were converted into one-dimensional intensity profiles as a function of the scattering vector q, I(q) vs. q, prior to data analysis.

## Results and discussion

**Fig. 3:**
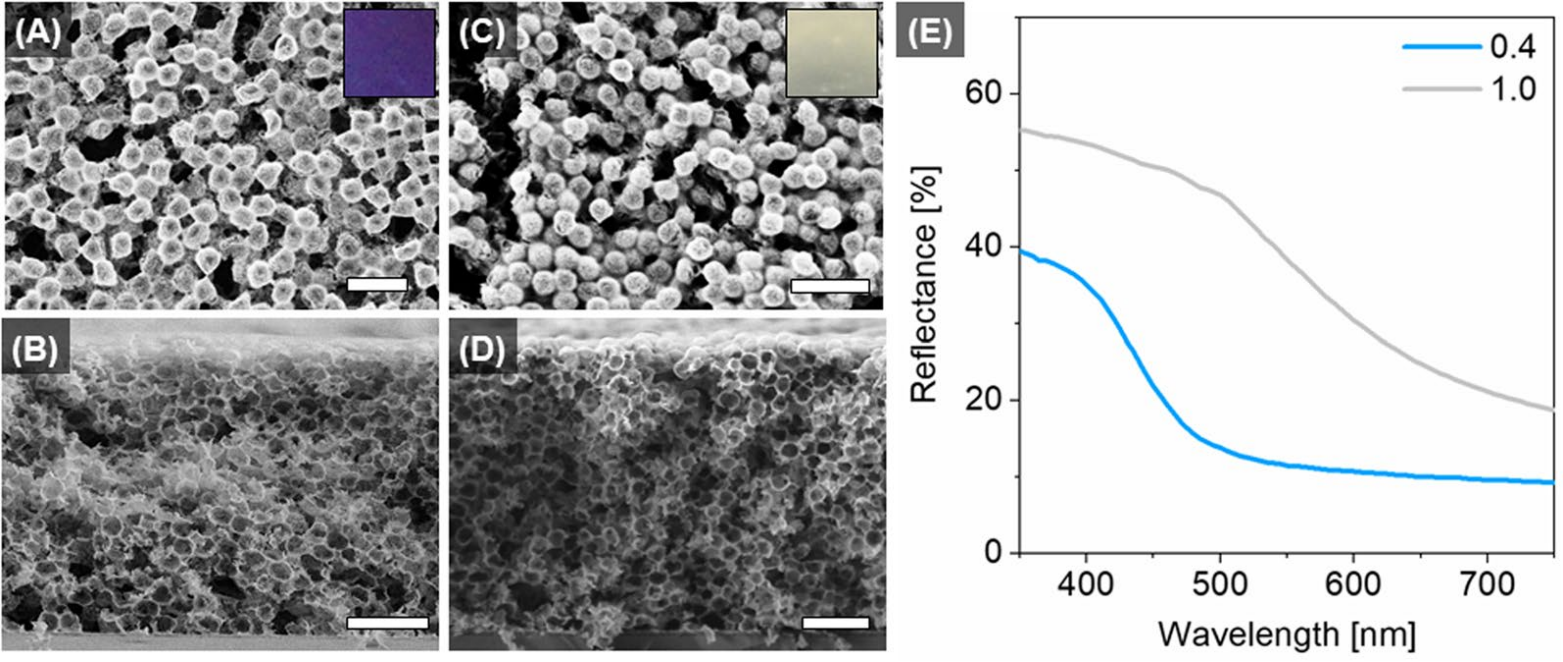
Morphology of hollow-sphere YSZ PhGs in (**A**, **C**) top view and (**B**, **D**) cross section imaged by SEM and (E) diffuse reflectance spectra of (**A**, **B**) 0.4-PhG and (**C**, **D**) 1.0-PhG ratios. The insets with 2.3 × 2.4 mm show the resultant structural color recorded at 0° angle of diffuse illumination on top of a black background. Scale bars correspond to 1 µm

The scanning electron microscopy (SEM) analysis of the top view and cross section of the PhGs (Fig. 3a–d) show that both types of PhGs, produced with YSZ/PS ratios of 0.4 and 1.0, presented a 3D disordered structured as expected from the heterocoagulation process. Even though the structural morphology of such PhGs seems similar, their optical properties are entirely different (Fig. 3e). An analysis of the diffuse reflection spectra in Fig. 3e reveals a sharp reflection edge with an inflection point at 425 nm of wavelength for the 0.4-PhG, while for the 1.0-PhG there is a flattening of the reflection edge resulting in a smoother transition from high reflectance at short wavelengths to low reflectance at longer wavelengths. In the short wavelength region of the visible spectrum, the PhG reflects at wavelengths corresponding to blue color, while in the long wavelength region the reflectance is relatively low. Due to the sharp reflection edge, i.e. steep decrease in the transition region, a highly saturated blue structural color arises for the 0.4-PhG. Meanwhile, for the 1.0-PhG, the less steep shifted reflection edge leads to a higher reflectance also of other wavelengths, giving rise to a perception of a mixture of colors, i.e. white structural color. By increasing the YZS/PS ratio, it is expected that the PhG final shell thickness will also increase, since there is a higher amount of YSZ per unit volume. It was therefore hypothesized that the thickness of the YSZ shell in the hollow-sphere PhG was the reason for the reduction of the reflection edge steepness and consequently also of the resultant color.

**Fig. 4:**
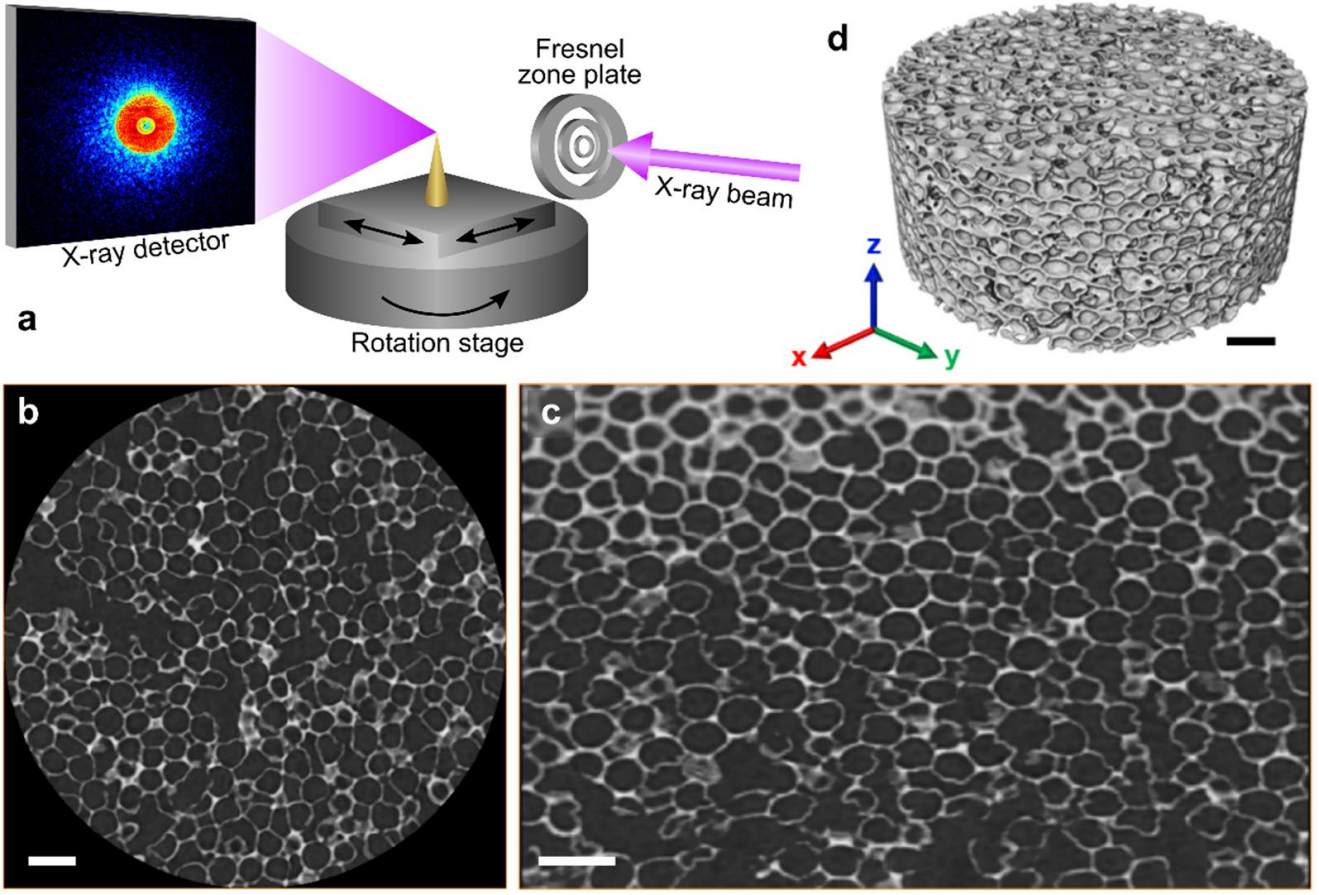
PXCT measurement and analysis: **a** schematic drawing of the measurement set-up used to achieve ultra-high resolution PXCT. Two-dimensional slices of the reconstructed data in **b** x–y direction and **c** y–z direction in relation to the pillar axis. The 3D rendering of slices results in a 3D structure, exemplified by **d**. Note that a volume inside the sample pillar was cropped out in **b** and **c**. The sample depicted in this image is the 0.4-PhG. Scale bars represent 500 nm

To assess this hypothesis, both samples were measured by PXCT and the resultant reconstructed slices (as the exemplary shown in Fig. 4) as well as the 3D rendering were analyzed in detail. Both YSZ-PhGs presented a complex 3D structure comprised of structural features in different shapes and sizes on several length scales ranging from nanoscale for the YSZ shells to sub-microscale for the macropores’ diameter and further to microscale regarding the PhG thickness. Figure 2c gives an overview of the structural features that are present in a model structure of a YSZ hollow-sphere PhG.

**Fig. 5:**
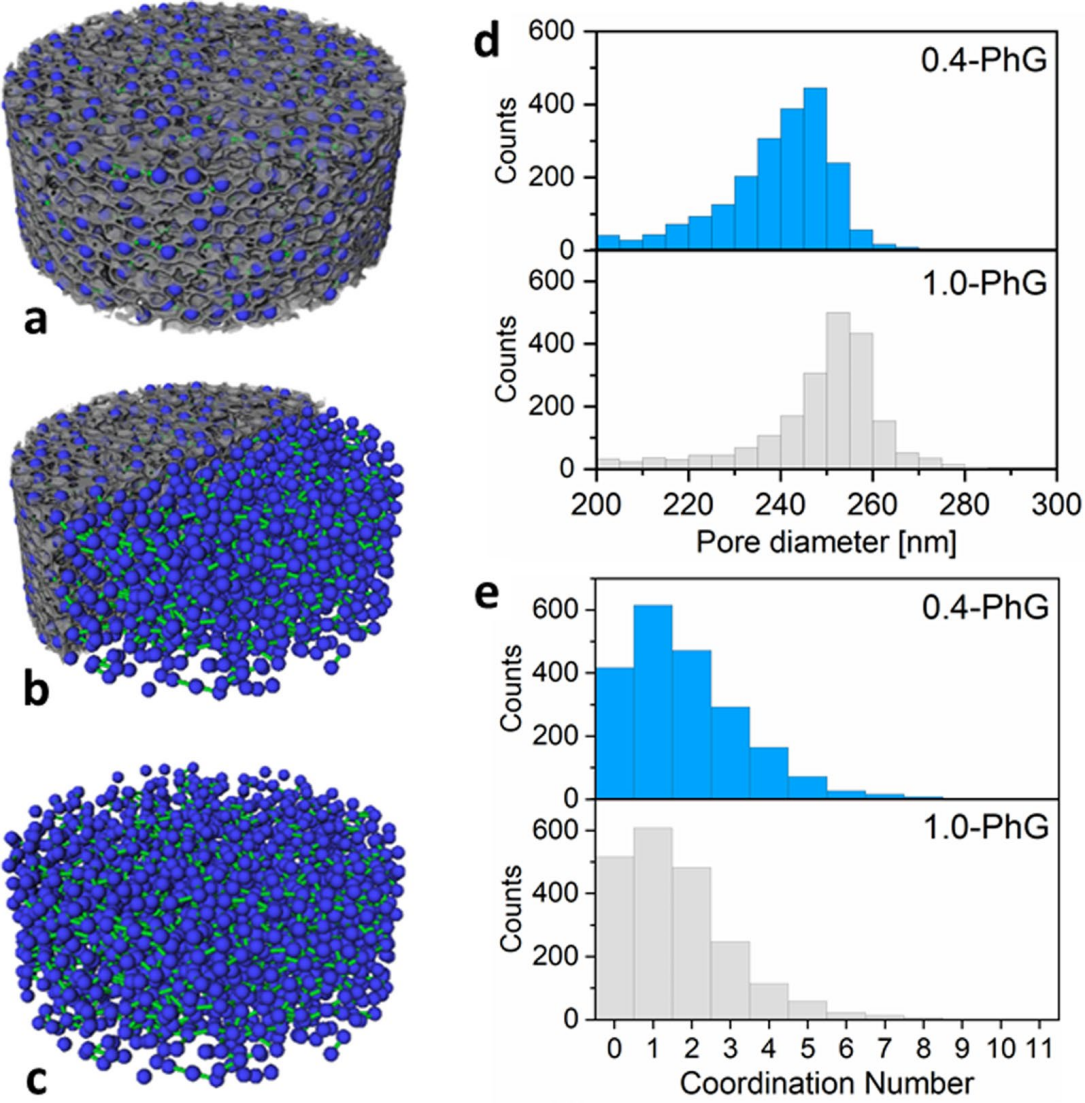
**a**–**c** Exemplary 3D reconstruction of the YSZ phase and pore network model for the 0.4-PhG sample. The macropores represented by blue blobs are connected through open sites within the YSZ shells. These connections are represented using green rods, while the solid YSZ phase is shown in grey color. The image in **b** has a crop of the YSZ phase volume rendering, while **c** shows only the pore network model without surrounding YSZ phase. The model is used to obtain the **d** macropores size and the **e** macropores’ coordination number distribution

The first difference identified between the two PhGs was the average macropore size (Fig. 5). In our YSZ-PhGs, the macropore size should be pre-defined by the PS template (nominal diameter 295 nm). Nonetheless, upon template burn-out (calcination), it is expected that the YSZ nanoparticles located at the shells start to sinter, leading to a global shrinkage of the 3D PhG. The macropore diameter size distribution (Fig. 5d) reveals a mean diameter of 239.3 ± 12.7 nm for 0.4-PhG and 248.8 ± 14.3 nm for 1.0-PhG, while the median value is 241.6 nm and 252.0 nm, respectively. Overall, the 1.0-PhG presented a slightly larger macropore size than the 0.4-PhG in a seemingly narrower size distribution.

The fact that the 1.0-PhG presents a smaller average shrinkage of 15% against 19% of the 0.4-PhG could be directly related to their sintering behavior. The shrinkage of the macropores is associated to the sintering of the YSZ nanoparticles that comprise the shell, which starts with the formation of necks between the YSZ particles and further densification associated with a reduction in the nanoparticle’s center distance. For the sample with less YSZ (0.4-PhG), the number of nanoparticles is smaller compared to the one with higher YSZ content (1.0-PhG), so one might infer that the shells of these samples have a different number of necks per volume due to the different number of particles per volume, but also the potential different packing fraction (later confirmed in the SAXS analysis). Nonetheless, following this assumption one would expect the samples with more potential necks (1.0-PhG) to shrink more than the sample with less necking (0.4-PhG), in the unrealistic case where both samples would have exactly the same packing at the shells. However, one must also account for the fact that the smaller packing directly relates to a smaller green density, which has been known and associated to higher shrinkage upon sintering under the same conditions when compared with samples of higher green density [37]. Moreover, thinner shells are hypothesized to be weaker than thicker shells, as in the case of atomic layer deposition (ALD)-based photonic crystals, and thus, less stable against cracking and distortions [38]. As a result, the macropores shrinkage is not only bigger, but more variations in the average diameter are observed for the 0.4-PhG compared to the 1.0-PhG. At last, the relative standard deviation associated to the macropore size increased from 3.1% (value given by PS supplier) to 5.7% (0.4-PhG) and 5.3% (1.0-PhG) indicating that the calcination step leads to an overall broadening of the macropore size distribution for both samples. This result correlate well with the observed optical properties (Fig. 3e), as it explains why the spectra is red-shifted.

Furthermore, the 3D distribution of the macropores was analyzed using a pore network model (Fig. 5c, e). The 3D distribution of the macropores and their coordination number can be indirectly associated to the PhG packing fraction [39, 40] (analyzed later), but also indicate whether there is the occurrence of ordered clusters inside the PhG structure. For an ordered FCC-like arrangement of the macropores, a coordination number of 12 is expected, while for random packing this number might vary between 4.5 and 9.0 [40, 41]. Hence, the coordination number distribution can be used as a means to evaluate disorder qualitatively when comparing pore networks. Note that for the pore network model analysis and the computation of the frequency distribution of the coordination number, we have only considered macropores and not voids (later described). Also, when a macropore is isolated (surrounded by voids or cracked areas), its coordination number will account to 0 since it has no connection to another macropore. The frequency distributions of coordination numbers of both samples resemble in shape and position, indicating that both samples exhibit a similar pore network structure with a similar degree of disorder. Moreover, the mean values for the coordination number are quite similar with 1.8 ± 1.6 and 1.6 ± 1.5 for 0.4-PhG and 1.0-PhG, respectively. Thus, the arrangement of the macropores and with that the packing of the hollow spheres is not affected by the increase of YSZ amount. Nonetheless, these low numbers were not only surprising, but also not fitting what was predicted in theory [40]. However, once examining the fabrication process closely, one can understand why such low coordination numbers were obtained: in a successful heterocoagulation process, the positively charged YSZ nanoparticles will surround the negatively-charged PS particles forming a “shell” around such particles before they assemble into the PhG structure. That means that the PS particles which are fully covered by YSZ nanoparticles will not touch and form connection points, since only their shells—or in other words the YSZ nanoparticles—will touch. This means that the low coordination numbers actually indicate a quite successful and stable heterocoagulation process, rather than an issue in the samples. Nonetheless, this fact hinders the estimation of the packing fraction based on the coordination number solely.

**Fig. 6:**
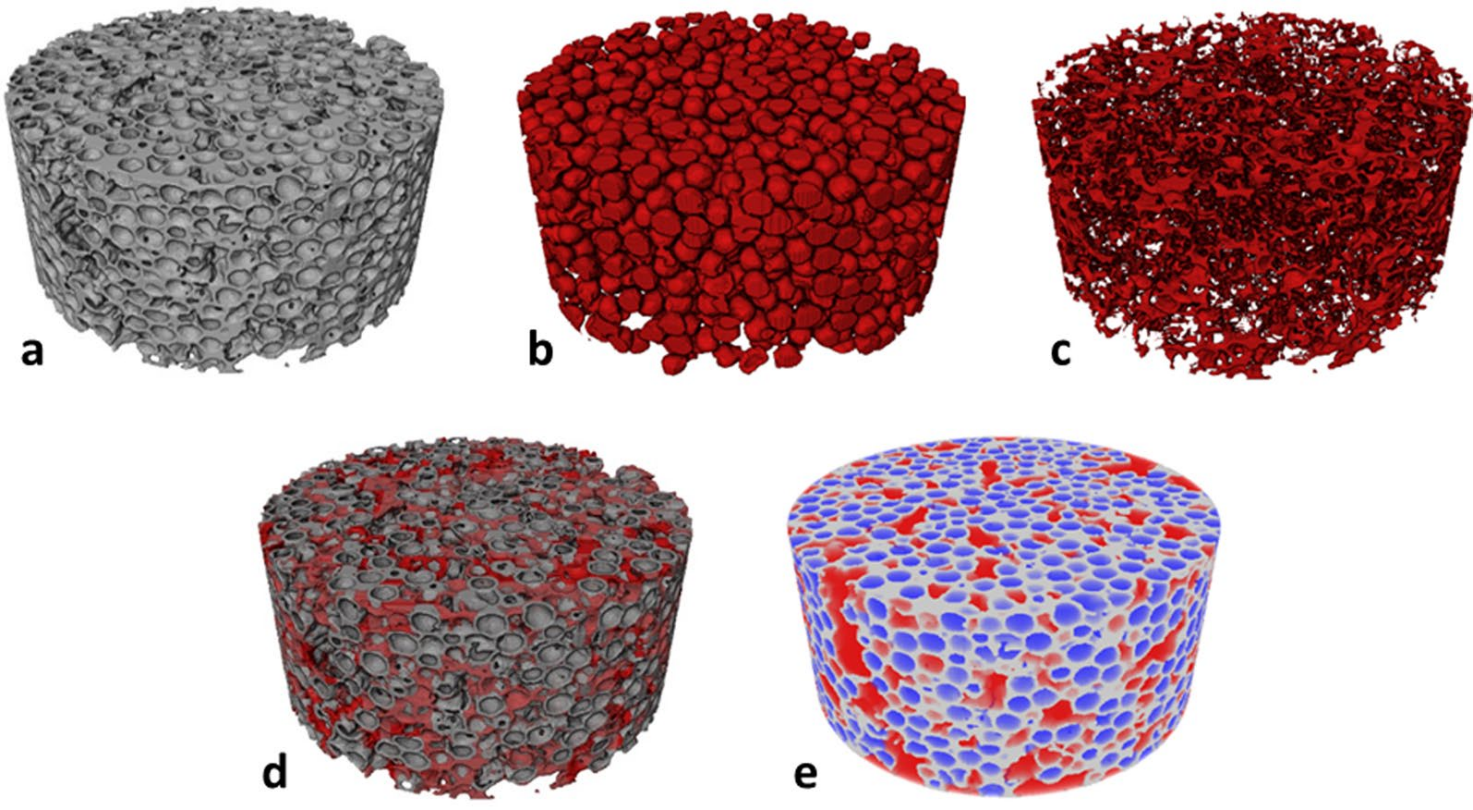
Exemplary 3D volume rendering of YSZ hollow-sphere 1.0-PhG (1.0 YSZ/PS) showing **a** YSZ phase, **b** macropores, **c** interstitial YSZ and **d** combination of YSZ shells and interstitial YSZ, **e** combination of YSZ phase, macropores and voids. Check the Supplementary Information for the exemplary Video S1 of a 3D structure containing these features

 The packing fraction can also be assessed by 3D volumetric phase fraction analysis (Fig. 6). We analyzed first the “packing” of the non-solid phase, air, which results in 69 and 65 vol% for 0.4-PhG and 1.0-PhG, respectively. As those values were larger than the maximum-possible packing fraction expected for a random packing (64 vol% [40]), we dug further into the phase analysis to understand why we were obtaining higher numbers than “physically possible”. A closer analysis revealed that from these air phase fraction percentages, only 34 and 36 vol% corresponded to macropores, i.e. pores that originate from burning out the PS template spheres (spherical air blobs surrounded by YSZ shells) and are responsible for the photonic response. It became clear that a phase differentiation must be made in between macropores and the remaining air “filled” volumes of the PhG, hereby called voids. In the case of our PhGs, four phases are then present: macropores and voids (air), YSZ shells and interstitial YSZ (solid phase), shown in the exemplary 3D renderings of Fig. 6. Further details are given in the Supplementary Information and Fig. 2c. This differentiation is made since voids are detrimental to the photonic glasses’ optical properties in the sense of spoiling the structural color. Although such voids could be expected from a drop-cast self-assembly process, it was surprising that the voids correspond to such a high-volume fraction of 35% and 29% for 0.4-PhG and 1.0-PhG, respectively. This phase fraction can be further subclassified into big voids (diameter > 300 nm) and small voids (diameter < 300 nm, sphericity < 0.7). Interestingly, the volume fraction of big voids was equal in both samples (24 vol%) while the volume fraction of small voids decreased with higher YSZ/PS ratio. We thus relate the big voids to the self-assembly process, since we have also observed those in a previous study [3].

Followingly, we analyzed the volume fraction of the solid phase, YSZ, in more detail. To distinguish between interstitial YSZ and YSZ shells, i.e., layer of YSZ that envelops the macropores, the following procedure was conducted: the whole solid phase (Fig. 6a) was subtracted from the macropores phase, which was dilated by 3 voxels to mimic the sample condition where macropores are surrounded by YSZ shells (Fig. 6b). Note that the voxel size is identical for both samples. The result of this 3D subtraction are irregular-shaped objects which correspond to the, hereby called, interstitial YSZ (Fig. 6c). A phase fraction analysis is then performed for both phases, visualized in the combined volume rendering of interstitial YSZ and YSZ shells shown in Fig. 6d. As expected, the determined volume fraction of YSZ shells and interstitial YSZ was smaller for the 0.4-PhG, accounting to 28 and 3 vol% against 30 and 5 vol% for the 1.0-PhG sample. The higher shell volume fraction hinted at a higher shell thickness.

**Fig. 7:**
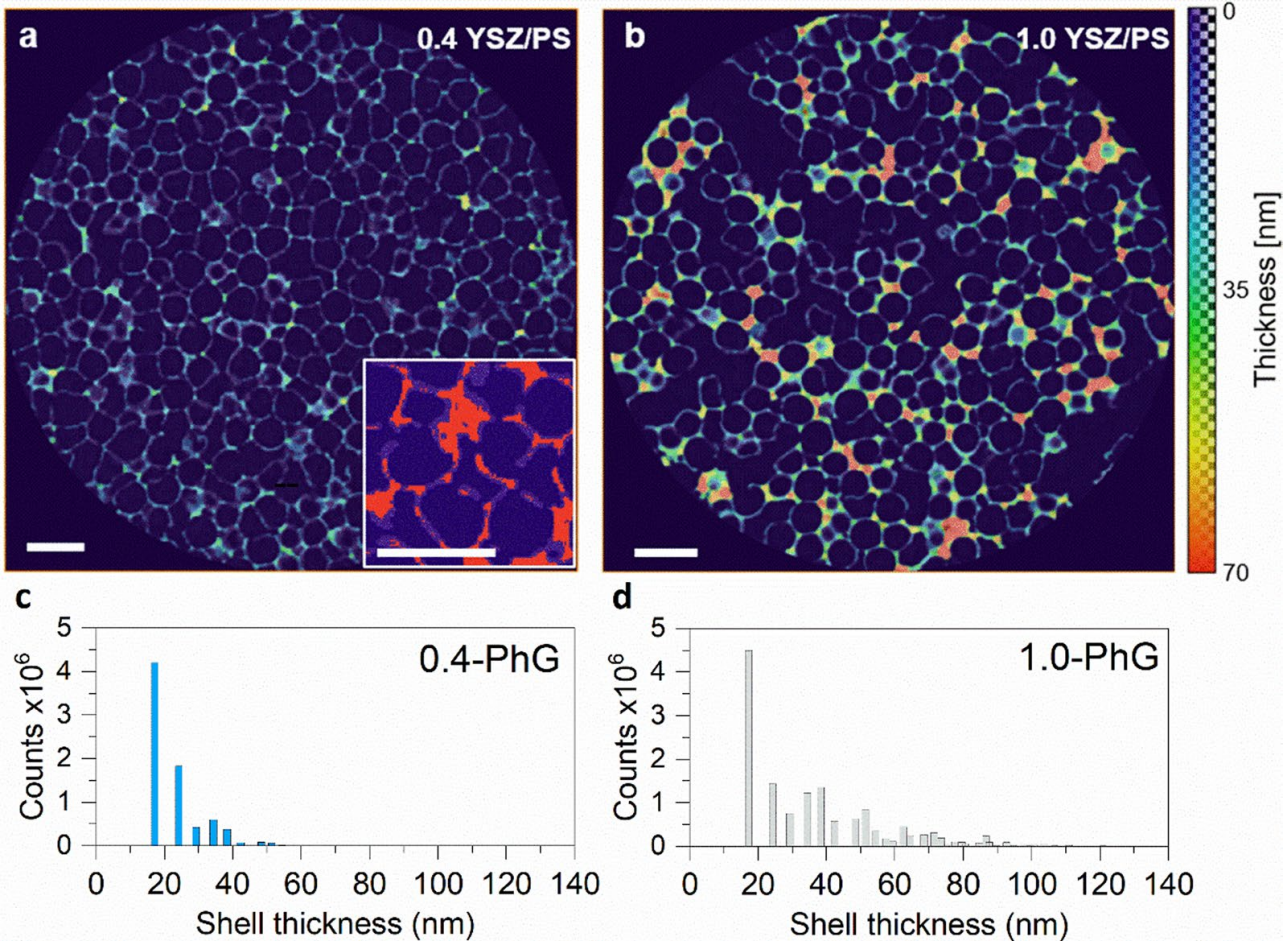
Exemplary YSZ phase thickness maps overlapped into xy-slices and their resulting histograms of the **a–c** 0.4-PhG and **b–d** 1.0-PhG samples. The local 3D thickness values of the YSZ phase are color coded according to the color scale shown on the right of the image. For example, red areas show the regions with highest local thickness while light blue indicates very thin regions of the YSZ phase. The inset in **a**, which is not color coded, shows the regions included by the thickness map in red, while the area that is assigned to the YSZ phase, but is not covered by the thickness map, is given in light purple. Dark purple regions represent the air phase. Scale bar is 500 nm

Thereby, phase thickness maps of the YSZ solid phase were created for both samples (Fig. 7). The computed thickness maps allowed the visualization of the three-dimensional local thickness of each voxel at its corresponding position within the selected phase, being color-coded according to the varying thicknesses. The thickness map of the 0.4-PhG sample shows mostly light blue coloring corresponding to thin shells, with thicker areas located either at triple macropore contact points or surrounding big voids. This can also be observed in the 1.0-PhG sample, albeit overall the color code points to only slightly thicker shells. Interestingly, the areas with most increased thickness are located in between macropores, reaching values up to around 70 nm. This corresponds to interstitial YSZ, explained in the previous paragraph. The thickness histograms (Fig. 7c–d) extracted from the values of the thickness maps provide an overview of the shell thickness values. Both histograms are cut at 17 nm, i.e. present 0 counts below this value, due to the detection limit associated with the measurement resolution. For the 0.4-PhG the distribution mean is centered at 23.8 nm, while the mean for the sample 1.0-PhG is centered at 39.2 nm. This much higher mean value—not “visualized” in the thickness map—is associated to the higher counts for values above 30 nm, clearly related to interstitial YSZ and not the shell.

Although the overall appearance of the thickness maps and histograms indicates the presence of thicker shells in the 1.0-PhG than in the 0.4-PhG sample, it is necessary to point out the limitations of the thickness map computations. During the results’ analysis, it became clear that the computed thickness map does not recognize (compute) all parts of the YSZ phase, especially not the thinner parts and borders of the shells (see inset in Fig. 7a). This is due to the limitation imposed by the PXCT measurement resolution. Even though the values for the estimated resolution were 8.9 nm and 10.5 nm with a pixel size of 8.6 nm, the obtained mean thickness values are quite close to the SEM-estimated shell thickness values around 20–30 nm. This means that for the thinnest shell, only 2–3 voxels would be representing it. Therefore, the computed thickness map “fails” at these locations, which may lead to inaccurate results for (very) low values and thus, should be used preferably for qualitative comparative analysis. With that in mind, both the thickness maps and the histograms point out to an increase of YSZ at the interstitial sites with the increasing YSZ/PS ratio, rather than a significant increase of the shell thickness in the final YSZ hollow sphere PhG structure.

**Fig. 8:**
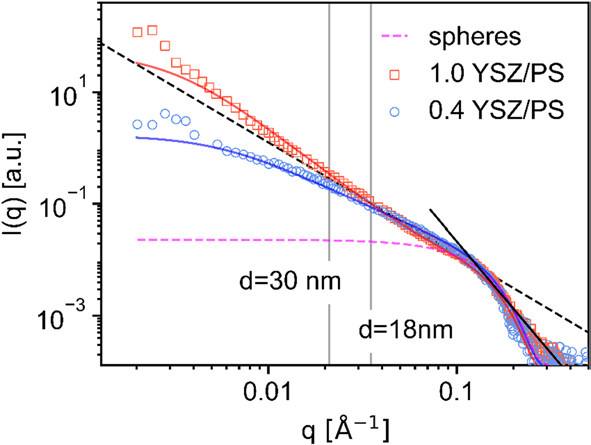
SAXS analysis of aqueous suspensions containing heterocoagulated YSZ-PS structures formed by YSZ nanoparticles’ shells and 295 nm-sized PS particles with the YSZ/PS ratio of (circular marker) 0.4 and (square marker) 1.0. I(q) is the intensity and q the scattering vector. The solid blue line and solid red line present the theoretical scattering curve of fractals comprised of spherical subunits. The lines present different slopes: q^−2^ (black dashed line) is characteristic for 2D planar objects (in this case the YSZ shell) and q^−4^ (black solid line) is typical for spherical objects (YSZ nanoparticles). Additionally, the scattering intensity of non-interacting spheres with a size of 3.0 ± 0.6 nm (Gaussian distribution) is given by the pink dashed line

Since this was an unexpected result for us, we have decided to further investigate the shell formation and thickness by analyzing the heterocoagulated structures by SAXS. Previous measurements by Retsch’s group [42] and Armes’ group [43, 44] have demonstrated that for sol–gel-based spherical core–shell particles and hollow silica particles with particulate shells, respectively, the border between the core and the shell can be linked to characteristic oscillations in the scattering data of SAXS measurements. Based on these previous works, SAXS measurements were conducted with suspensions of heterocoagulated YSZ nanoparticles and PS template spheres representing the state of the shells before removal of the PS templates via calcination, i.e. heterocoagulated core–shell PS-YSZ structures formed by the oppositely charged particles (Fig. 8**)**.

Both suspensions presented the same power laws at the high *q*-region (*q*^−4^ for *q* > 0.13 Å^−1^), which describes their smallest structural feature corresponding mainly to single YSZ nanoparticles. Their size, d, determined by the change in slope accounts to approximately 4 nm confirming the size obtained from SAXS analysis of the YSZ nanoparticles’ stock suspension in our previous work, which was 4.3 ± 1.6 nm [8]. At a very zoomed view, i.e. on a small scale, the PS-YSZ core–shell spherical heterocoagulated structures present only a small local curvature, therefore, the shell can be described as a 2D structure comparable to a thin flat sheet [42]. Strong scatterers with mainly 2D structures will result in SAXS data following the *q*^−2^ power law (black dashed line in Fig. 8) which in our measurements applies to the range of ca. 0.03 Å^−1^ < *q* < 0.13 Å^−1^. Both samples presented similar scattering spectra at this intermediate *q*-region, thus, suggesting a similar shell morphology with a similar nanoparticulate shell texture. Nevertheless, the deviation from the *q*^*−2*^ scaling at intermediate *q* law hints to a correlation length inside the sample, which most probably is caused by an averaged YSZ thickness. Conversely to the sol–gel-based core–shell structures, the SAXS data of the heterocoagulated suspensions did not exhibit oscillations over the whole scattering vector (*q*) range, thus, indicating that the YSZ shells are less dense than a sol–gel generated shell. This is not surprising as the heterocoagulated shells are formed by YSZ nanoparticles, thus presenting a non-dense arrangement of particles. Using the estimation* d* = 2π/*q* results in thicknesses of approximately 18 nm and 30 nm for the suspensions with YSZ/PS ratio of 0.4 and 1.0, respectively, showing that indeed the shell thickness of 1.0 YSZ/PS is higher than 0.4 YSZ/PS.

Zooming in even more to the inside of the heterocoagulated nanoparticulate shells, their rough surface can be described in a simple approach as a fractal, [44] which is basically a cluster formed by spherical subunits with radius *r* and a correlation length. Fitting the model of non-interacting spheres to the change in slope at higher *q* results in spherical subunits of 3.0 ± 0.6 nm size (pink dashed line in Fig. 8) and using the estimated values of 18 nm and 30 nm for the correlation length, the scattering of the heterocoagulated YSZ/PS can be well described by the theoretical scattering of fractals (solid blue and red lines). Correlating these results with our processing method, indicates that clusters of YSZ nanoparticles were formed in the suspension before being adsorbed to the PS particle surface rather than a uniform adsorption of single YSZ nanoparticles to the PS surface (see schematic drawing in Figure S4), forming fractal-like shells. In this case, the fractal dimension *D*_*f*_ can be used to describe the packing fraction of the spherical subunits within the clusters (shells) and its value should increase as the packing density increases. The comparison between the experimental data and the fractal model yields a higher value of *D*_*f*_ = 2.45 for 1.0 YSZ/PS than *D*_*f*_ = 1.75 for 0.4 YSZ/PS. This means that for the 0.4 YSZ/PS, a loosely packed shell with smaller number of clusters is formed in comparison to 1.0 YSZ/PS, i.e. the shell of 1.0-PhG is more densely-packed. Such initial packing differences explain the different previously-discussed shrinkage behavior.

Nonetheless, the differences between the results for the YSZ shell thickness from SAXS and the findings from PXCT analysis could also point out to potential structural changes during processing, such as sintering of the YSZ nanoparticles during the burn-out of the PS spheres. The SAXS results are based on a correlation length of approx. 30 nm for the 1.0 YSZ/PS suspension, which would be possible to be resolved by the thickness map of the PXCT data. However, the shell thickness histogram without interstitial YSZ extracted from the PXCT thickness map of the 1.0-PhG was similar to the 0.4-PhG (Fig. 7), but it also showed the YSZ solid phase with higher thickness at the interstitial sites. This structural difference between the suspended heterocoagulated YSZ and PS particles and the final YSZ hollow sphere PhG is associated to the template removal step when the PhG is calcinated at 500 °C. It is well known that ceramic nanoparticles exhibit lower sintering temperatures compared to micronsized powders [37, 45] and the YSZ shells are formed by tiny YSZ nanoparticles with average size of ⁓ 4 nm. Early studies have shown that pronounced sintering already occurs below 600 °C for 6 nm-size ZrO_2_ nanoparticles and 4 nm-size Y_2_O_3_ nanoparticles [46, 47]. Similar results were obtained by Trunec and Maca [48] for YSZ compacts for which a relative density increase below 600 °C was only observed for the powder with the smallest average particle size of 10 nm. It is also known that sintering of powders starts with neck formation, followed by neck growth due to mass transport and densification and/or coarsening [45]. Thus, it is reasonable to assume that in a volume where more powder particles are present in contact, more necks will form compared to volumes with less contacts. These correspond to the interstitial sites in our PhG structures. It is possible then, that material will diffuse from the shells to the interstitial areas causing an apparent thinning of the shells. Similar observations were reported by Sokolov et al*.* [49] in highly-porous sol–gel-based aluminum oxide structures.﻿

These significant structural changes within the YSZ phase reveal that the YSZ hollow sphere PhGs deviate from the modeled hollow sphere PhG system that describes a disordered arrangement of mono-sized hollow spheres with defined shells, thus, the modeled structure not only assumes a much higher packing, but also does not consider the existence of interstitial YSZ. Despite the structural deviations between the PhG model and the experimental PhG, the experimental optical results are in good agreement with simulations [8]. However, based on the findings from our detailed PXCT analysis combined with SAXS, the smoothing of the reflection edge in the experimental case is most likely related to multiple scattering caused by the interstitial YSZ rather than the slightly increased shell thickness. The volumes occupied by interstitial YSZ phase promotes incoherent scattering, enhancing the “background” in the reflection measurements especially at longer wavelengths. This counteracts the selective scattering of blue light from the shells and, combined with lower packing, reduces color saturation.

## Conclusion

A detailed multi-scale structural characterization of ceramic-based PhGs with different YSZ nanoparticles’ amount was presented in this work. The results showed that the “real” YSZ hollow-sphere PhGs deviate from the modeled hollow-sphere PhG system, which describes a disordered arrangement of macropores with “solid” shells, assuming a much higher packing, but also ignoring the existence of interstitial YSZ. Based on the findings from our detailed PXCT analysis combined with SAXS, the smoothing of the reflection edge in the experimental samples is most likely related to the interstitial YSZ promoting incoherent scattering rather than just the slightly increased shell thickness, disproving our initial hypothesis.

The results obtained in this work point out to two major improvement points for the PhG-community. First, future PhG scattering models must consider different parameters for the simulations of optical properties rather than the “ideal PhG” or use imaging data to generate their structural models. Concomitantly, experimentalists need to revise, adapt and further develop their fabrication procedures to try to improve packing and reduce the number of voids in PhGs, which should lead to a better structural color, and to assure better control over desired structural parameters. At last, the authors consider that collaborative efforts between theoreticians and experimentalists will lead to faster advances in the photonics community.

### Supplementary Information

Additional file 1.

Additional file 2

Additional file 3.

## Data Availability

The data that support the findings of this study are available from the corresponding author, K.P. Furlan, upon reasonable request.
