# Supplementary material for: Multi-scale structural characterization of ceramic-based photonic glasses for structural colors
Source: Discov Nano. 2024 Jul 8;19(1):114. doi: 10.1186/s11671-024-04057-x (PMC11231108; doi:10.1186/s11671-024-04057-x)
Supplement: Supplementary file 1 — Additional file 1. [file 11671_2024_4057_MOESM1_ESM.pdf]

Supplementary Information

of

Multi-Scale Structural Characterization by PXCT and SAXS of Ceramic-Based Photonic Glasses for Structural Colors

Yen Häntsch[a], Ana Diaz[b], Mirko Holler[b], Tobias Krekeler[c], Martin Ritter[c], Sabine Rosenfeldt[d], Markus Retsch[d], Kaline P. Furlan[a,e]

a Hamburg University of Technology, Institute of Advanced Ceramics, Denickestraße 15, 21073 Hamburg, Germany

b Paul Scherrer Institute, Forschungsstrasse 111, 5232 Villigen PSI, Switzerland

c Hamburg University of Technology, Electron Microscopy Unit, Eißendorfer Straße 42, 21073 Hamburg, Germany

d University of Bayreuth, Department of Chemistry and Bavarian Polymer Institute, Universitaetsstr. 30, 95447, Bayreuth, Germany

e Hamburg University of Technology, Institute of Advanced Ceramics, Integrated Materials Systems Group, Denickestraße 15, 21073 Hamburg, Germany

1. 1. FIB preparation of PXCT pillars

The PhG structure was coated with a carbon protection layer prior FIB milling which can be seen in both SEM images as a dense layer on top of the hollow-sphere structure. The carbon layer was deposited to prevent the PhG film surface from damage induced by the FIB milling. Further, **Figure S1**a show a residual piece of the tungsten needle on top of the carbon layer, which was used to transfer the pillar from the silicon wafer to the tomography pin. Due to possible interaction and scattering of the X-ray beam at the tungsten piece, the top of the pillar was cleaned, and the final samples had a smooth surface with a 477 – 548 nm thick layer of carbon as shown in Figure S1b.

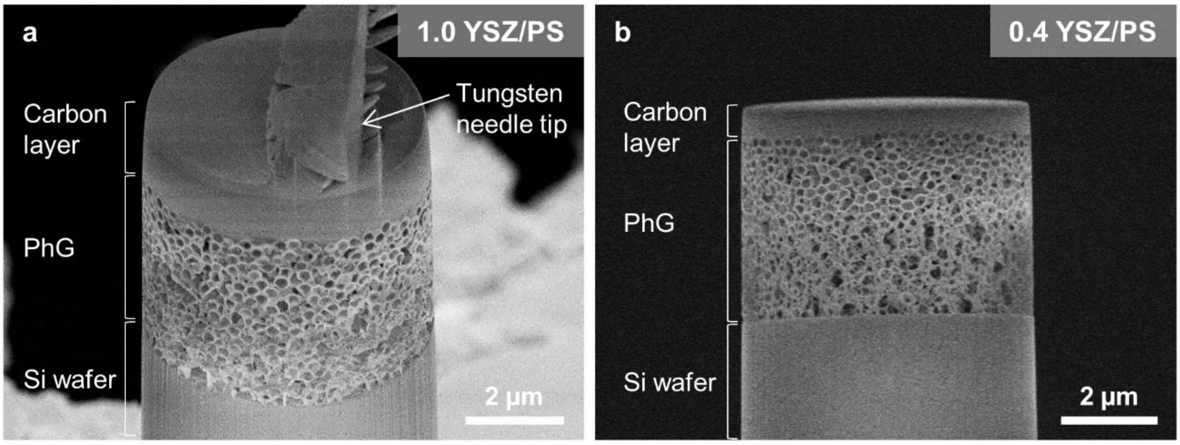

Figure S1. SEM images of cylindrical samples prepared via FIB for PXCT measurements. A protection layer of carbon was deposited on top of the PhG film. YSZ hollow-sphere PhGs were fabricated with 295 nm PS template spheres and a) 1.0-PhG and b) 0.4-PhG, respectively.

2. 2.PXCT measurements and data processing

Measurements were performed at the cSAXS beamline of the Swiss Light Source using a photon energy of 6.2 keV and a setup optimized for high-resolution PXCT.[1] The coherent illumination on the sample was defined by focusing the beam with a Fresnel zone plate (FZP) of 170 μm diameter and 60 nm outer-most zone width, which had a focal length of 51 mm at this energy, producing a beam with a flux of about 3.5×10^8 photons/s. The FZP, which was fabricated at the X-ray Nano-Optics group at the Paul Scherrer Institute in Villigen, Switzerland, had especially designed aberrations to obtain a structured illumination on the sample optimized for X-ray ptychography.[2] The sample was placed at 0.65 mm downstream of the focus, where the beam had a diameter of about 1.7 μm. Ptychographic scans of the sample were performed following the pattern of a Fermat spiral[3] with an average step size of 0.4 μm. At each scan position, coherent diffraction patterns with an acquisition time of 0.1 s were recorded with an Eiger 1.5M[4] detector developed by the detector group at the Paul Scherrer Institute. Ptychographic scans had a field of view of 8.5 μm × 6 μm and 10 μm × 7 μm (horizontal × vertical) for the 0.4 PhG and the 1.0 PhD samples, respectively. We repeated ptychographic scans at equally spaced rotation angles ranging from 0 to 180 deg following a non-sequential order, in such a way that a full tomogram is decomposed in 8 subtomograms with 8 times larger angular spacing, acquired subsequently.[5] In this way we could monitor changes in the sample due to X-ray radiation. We observed that the samples were changing slightly in the beam during acquisition, and stabilized after absorbing an estimated dose of about 2×10^9 Gy and 1×10^8 Gy for samples 0.4 PhG and 1.0 PhG, respectively. After stabilization, we recorded a full tomogram for each sample with 1100 equally angular spaced projections, for which we deposited an estimated dose of about 4×10^9 Gy. The dose was estimated as the surface dose, $D_s = \mu N_0 E/\rho$,[6] where N_0 is the number of photons per unit area incident on the sample, E is the photon energy, and μ and ρ are the linear attenuation coefficient and the density of the material. In our estimation we used zirconia for the material. The tomographic acquisitions took about 11.6 and 16.5 hours for each sample, including the acquisition time and the time between acquisitions due to the movement of stages.

Ptychographic reconstructions were performed with the PtychoShelves software package[7] developed by the Coherent X-ray Scattering group at the Paul Scherrer Institute, using 500 iterations of a maximum-likelihood algorithm working in GPUs with compact positioning grouping.[8] In the reconstruction we used the diffraction patterns cropped to a size of 700 × 700 pixels, resulting in a pixel size of 8.62 nm in the reconstructed images. The phase images were further processed to remove zero and first orders, and registered with subpixel accuracy[9,10] before being used for tomographic

reconstruction with filter back projection using a ram-lak filter with a cutoff frequency of 1. The 3D resolution of the resulting datasets was estimated by Fourier shell correlation to be to 8.9 nm and 10.5 nm for 0.4-PhG and 1.0-PhG, respectively. For this estimation we computed the Fourier correlation between two subtomograms, each acquired with half the angular projections, and compared it with the ½ bit threshold, as described in the paper by van Heel *et al.*. [11]

3. 3. Image analysis detailed procedure

First, the raw image was segmented into YSZ phase and air phase by manually selecting a specific grayscale value with interactive thresholding. Due to the big density difference between the YSZ phase and the air phase, interactive thresholding was straightforward and feasible. Assisted by an instantaneous visual feedback which presents selected pixels in blue color, the gray level threshold value was carefully chosen in such a manner, that all the pixels of darker areas were collected and identified as air phase. With that, all gray levels above this value were assigned to the YSZ phase while lower gray levels corresponded to the air phase. After image segmentation a 3D volume rendering of the reconstructed YSZ hollow-sphere PhG was obtained for quantitative 3D analysis of the structure. Based on the YSZ phase, the shell structure and volume fraction of YSZ was analyzed. To analyze the pores, the air phase was separated into individual objects using the watershed algorithm.[12]

Each of those individual objects possesses a three dimensional surface area A_{3D} and a specific volume V. Based on these two quantities, the diameter d and the sphericity S of an individual object, such as a macropore, were calculated via $d = \sqrt[3]{6 \times V/\pi}$ and $S = \pi^{1/3} \times 6V^{2/3}/A_{3D}$.The separated objects of the air phase were filtered by size, i.e. diameter, and by sphericity to distinguish between macropores and voids. Macropores are defined as pores that originate from burning out the PS template spheres, while voids correspond to the other remaining air-filled volumes of the PhG. Objects that fulfilled the criteria of sphericity > 0.7 and size between 200 nm and 300 nm were classified as macropores while all the other objects of the air phase that did not meet both specifications were assigned to be voids. The voids were further differentiated into small voids (size < 300 nm) and big voids (size ≥ 300 nm). Note that the upper limit of the macropore size was selected based on the nominal PS template size, which predefines the maximum-possible macropore size. The lower limit was defined based on previous studies of nanoparticulate YSZ photonic crystals,[13] while also accounting for the broader macropore size distribution. By that, the volume fraction of voids and macropores was estimated and characteristics of the macropores such as size distribution and pore network structure could be analyzed.

In order to determine the volume fraction of YSZ located at interstitial sites (so-called interstitial YSZ), which should not be accounted as a shell, the macropores were expanded by 3 pixels to mimic the macropores plus shells. The YSZ volume fraction, which was then located outside of these expanded macropores was assigned to interstitial YSZ and was assessed by subtracting the expanded macropores from the YSZ phase.

For the creation of thickness maps, at each voxel position, a ball with the largest diameter which fits inside the selected object (YSZ solid phase) and is centered by the respective voxel, was computed by the 3D image analysis software. The thickness values of the map are corresponding to the diameter of the computed balls. Thereby, the thickness map displays the spatially resolved values for the YSZ shell thickness for each xy-slice, being the one shown in Figure 8 an exemplary slice.

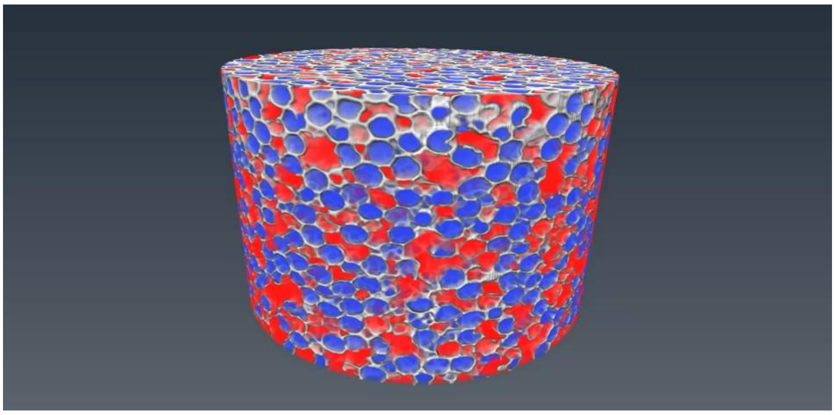

Video S1. Animated 3D rendering of the PXCT tomograms from the YSZ hollow-sphere PhG, showing the structural features quantified in the image analysis, color coded as (grey) shell thickness, (blue) macro pores, and (red) voids.

4. 4. Definition of the Volume of Interest: Analysis of the effect of carbon deposition

Before proceeding with quantitative analysis of the PhGs' features, it is necessary to choose a volume of interest (VOI) which is representative for the whole sample. Thereby, a preliminary analysis of the whole dataset, i.e. all slices obtained during the PXCT measurement, was performed and thoroughly evaluated. The volume fraction analysis per slice with thickness of a single voxel (8.62 nm) in xy-plane gives information about the material distribution related to the sample height (**Figure S2**a), where slice number 0 denotes the tomographic slice at the bottom of the 3D PhG which sits on top of the substrate. With rising slice number, the slice is located closer to the surface of the film which is covered by a carbon protection layer from the FIB sample preparation. In both samples, a distinctive drop of the air phase volume fraction and a simultaneous increase of the YSZ phase volume fraction was observed after slices 245 (0.4-PhG) and 270 (1.0-PhG). This observation is

assumed to be connected to the deposition of the protective carbon layer needed for FIB preparation and an unwanted carbon "infiltration" of the YSZ hollow sphere structure to a certain depth, since no inhomogeneity was observed for the distribution of YSZ nanoparticles. The assumption of carbon infiltration is further supported by the appearance of isolated macropores within the 3D sample reconstruction (Figure S2c).

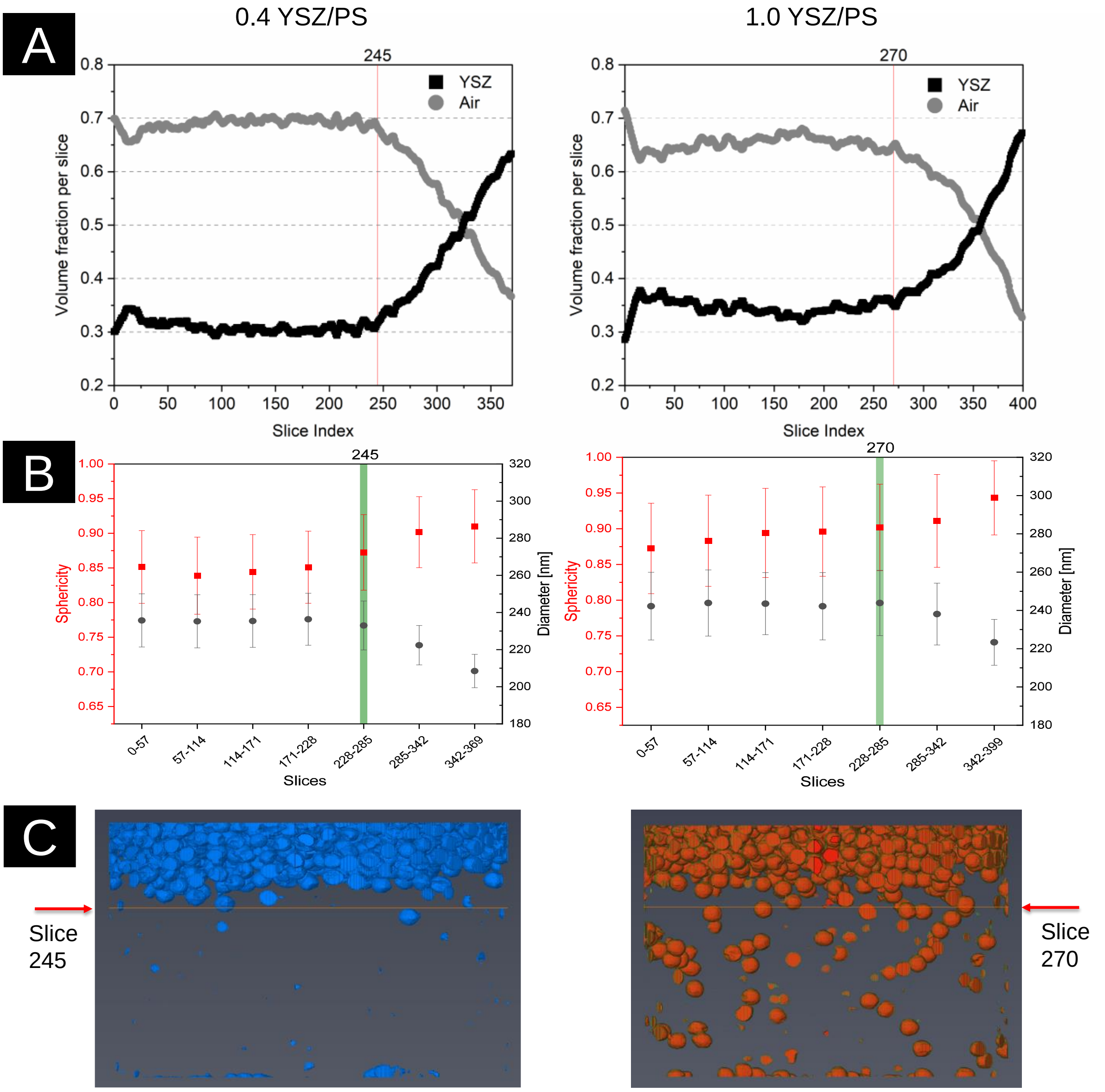

Figure S2. 3D analysis' results showing the carbon infiltration within the sample originating from FIB preparation (a) 3D volume fraction, (b) sphericity, (c) isolated macropores. Such carbon layer is necessary to avoid charging and mechanical drift during FIB milling and the absence of such layer hinders the preparation of perfectly circular pillars, as well as cause the distortion of the PhG structure due to excessive local heating caused by the beam. The common platinum cannot be used in this case because of its high absorbance of X-ray radiation, which would hinder the achievement of ultra-high resolution. Conversely, carbon is almost 'invisible' to the radiation under the conditions

used in the PXCT experiment. The red lines show the cut-off value for the slices considered in the 3D quantification of features of the PhGs, i.e. slides above such line were not included.

Interestingly, both samples present a distinct region in the upper part of the sample exactly above slices 245 (0.4-PhG) and 270 (1.0-PhG). Below this region, only a relatively low number of isolated macropores are found, which are then indeed related to YSZ agglomerates. Furthermore, the macropores' sphericity and diameter were analyzed by sectioning the sample along the z-axis, i.e. the sample height (Figure S2b). Each section contained 58 consecutive xy-slices with single voxel thickness for each slice, which is equal to a section with approx. 500 nm height. In both samples, the sphericity increases for the sections located closer to film surface while the diameter is decreasing. Considering slices 245 (0.4-PhG) and 270 (1.0-PhG) as the last slices without carbon infiltration, the changing values for sphericity and diameter start occurring within the sections that contain the slices where the volume fraction of the air phase drastically decreases (Figure S2a). This indicates that the increasing sphericity and decreasing macropore diameter are caused by the carbon deposition while carbon presumably deposits at rough and uneven sites inside the hollow spheres, thereby smoothening the macropore structure while reducing its diameter.

Another finding that supports the hypothesis of the carbon infiltration is the decrease of the macropores' diameter closer to the film's surface (Figure S2b), better visualized in the thickness map of the solid phase in z-direction, which enables the representation of 3D local thickness at each point within the selected object or feature (**Figure S3**). The regions with high local thickness values of approx. 70 nm are marked in red while for thickness values below approx. 30 nm respective areas are highlighted in light blue. The thickness map on the left side (Figure S3a) is located in the region without carbon infiltration whereas the thickness map on the right side (Figure S3b) was extracted from the upper part of the sample where carbon has infiltrated the structure. Comparison between the thickness maps reveals that the shells of the upper slices are notable thicker due to the carbon infiltration. Moreover, there was no indication for inhomogeneous heterocoagulation during the fabrication, no aspects that could indicate sample modification during FIB and no observation of irregular YSZ regions in the SEM cross-section analysis along the film height, all supporting the conclusion of the carbon infiltration. Based on these findings, the volume of interest was defined to be comprised by slices 0 – 245 and 0 – 270 for 0.4-PhG and 1.0-PhG, respectively. With that, 124 and 129 slices were excluded resulting in an estimated carbon infiltration depth of around 1 μm considering the edge length of a cubic voxel of 8.62 nm. This information is of relevance for future FIB sample preparations for PXCT measurements.

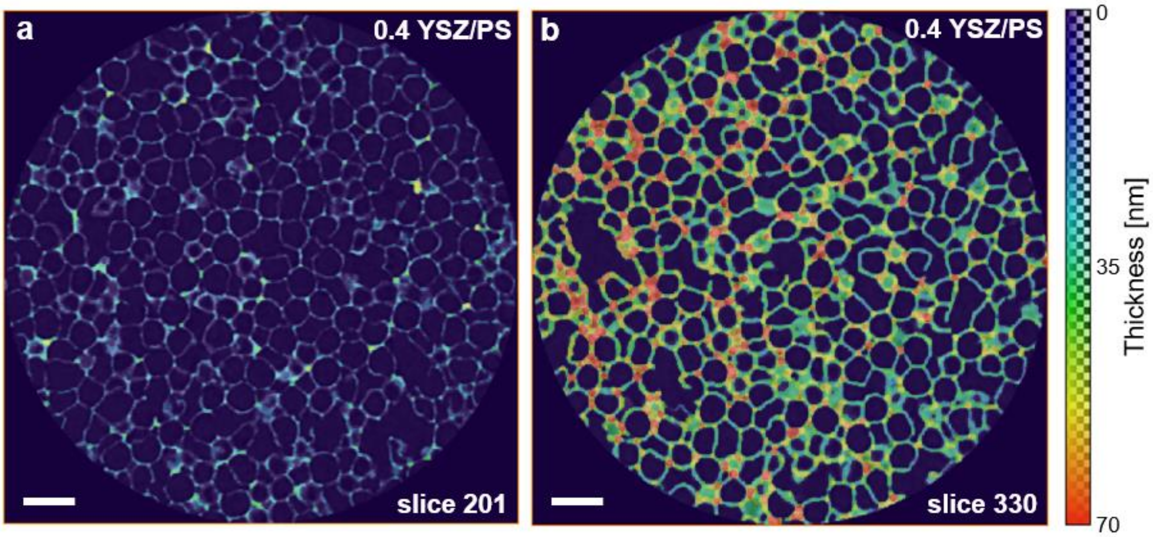

Figure S3. Computed thickness maps of the 0.4-PhG solid material phase. Thickness maps were extracted at different film heights: a) lower part of the film without carbon deposition at slice no. 201 and b) upper part of the film infiltrated with carbon at slice no. 330. The color scaling shows visually the increased thickness of the YSZ phase in the upper part of the film. Scale bar is 500 nm.

5. 5. SAXS analysis

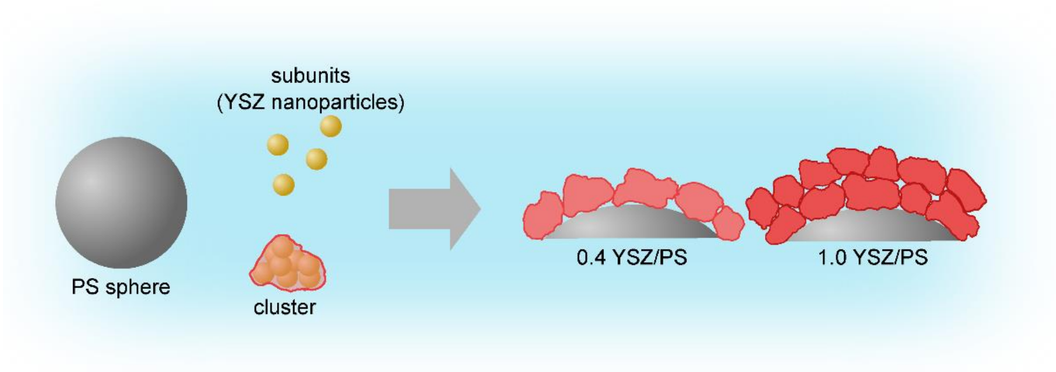

Figure S4. Schematic drawing of the interpretation of the fractal model applied to the heterocoagulated YSZ nanoparticles and the PS spheres forming a core-shell structure. The clusters (fractals) consist of YSZ nanoparticles aggregating in the suspension. The clusters (represented in red) adsorb at the PS surface. The higher packing density of the clusters is represented by the more saturated red color.

6. References used in this section

1. Holler, M.; Diaz, A.; Guizar-Sicairos, M.; Karvinen, P.; Färm, E.; Härkönen, E.; Ritala, M.; Menzel, A.; Raabe, J.; Bunk, O. X-ray ptychographic computed tomography at 16 nm isotropic 3D resolution. *Sci. Rep.* **2014**, *4*, 3857 EP -, doi:10.1038/srep03857.

2. Odstrčil, M.; Lebugle, M.; Guizar-Sicairos, M.; David, C.; Holler, M. Towards optimized illumination for high-resolution ptychography. *Opt. Express* **2019**, *27*, 14981–14997, doi:10.1364/OE.27.014981.
3. Huang, X.; Yan, H.; Harder, R.; Hwu, Y.; Robinson, I.K.; Chu, Y.S. Optimization of overlap uniformness for ptychography. *Opt. Express* **2014**, *22*, 12634–12644, doi:10.1364/OE.22.012634.
4. Dinapoli, R.; Bergamaschi, A.; Henrich, B.; Horisberger, R.; Johnson, I.; Mozzanica, A.; Schmid, E.; Schmitt, B.; Schreiber, A.; Shi, X.; et al. EIGER: Next generation single photon counting detector for X-ray applications. *Nucl. Instrum. Methods Phys. Res., Sect. A* **2011**, *650*, 79–83, doi:10.1016/j.nima.2010.12.005.
5. Münch, B. Spatiotemporal computed tomography of dynamic processes. *Opt. Eng* **2011**, *50*, 123201, doi:10.1117/1.3660298.
6. Howells, M.R.; Beetz, T.; Chapman, H.N.; Cui, C.; Holton, J.M.; Jacobsen, C.J.; Kirz, J.; Lima, E.; Marchesini, S.; Miao, H.; et al. An assessment of the resolution limitation due to radiation-damage in x-ray diffraction microscopy. *J. Electron Spectros. Relat. Phenomena* **2009**, *170*, 4–12, doi:10.1016/j.elspec.2008.10.008.
7. Wakonig, K.; Stadler, H.-C.; Odstrčil, M.; Tsai, E.H.R.; Diaz, A.; Holler, M.; Usov, I.; Raabe, J.; Menzel, A.; Guizar-Sicairos, M. PtychoShelves, a versatile high-level framework for high-performance analysis of ptychographic data. *J. Appl. Crystallogr.* **2020**, *53*, 574–586, doi:10.1107/S1600576720001776.
8. Odstrčil, M.; Menzel, A.; Guizar-Sicairos, M. Iterative least-squares solver for generalized maximum-likelihood ptychography. *Opt. Express* **2018**, *26*, 3108–3123, doi:10.1364/OE.26.003108.
9. Guizar-Sicairos, M.; Diaz, A.; Holler, M.; Lucas, M.S.; Menzel, A.; Wepf, R.A.; Bunk, O. Phase tomography from x-ray coherent diffractive imaging projections. *Opt. Express* **2011**, *19*, 21345–21357, doi:10.1364/OE.19.021345.
10. Odstrčil, M.; Holler, M.; Raabe, J.; Guizar-Sicairos, M. Alignment methods for nanotomography with deep subpixel accuracy. *Opt. Express* **2019**, *27*, 36637–36652, doi:10.1364/OE.27.036637.
11. van Heel, M.; Schatz, M. Fourier shell correlation threshold criteria. *J. Struct. Biol.* **2005**, *151*, 250–262, doi:10.1016/j.jsb.2005.05.009.
12. Soille, P. *Morphological Image Analysis;* Springer Berlin Heidelberg: Berlin, Heidelberg, 2004, ISBN 978-3-642-07696-1.
13. Kubrin, R.; do Rosario, J.J.; Lee, H.S.; Mohanty, S.; Subrahmanyam, R.P.; Smirnova, I.; Petrov, A.; Petrov, A.Y.; Eich, M.; Schneider, G.A. Vertical convective coassembly of refractory YSZ inverse opals from crystalline nanoparticles. *ACS Appl. Mater. Interfaces* **2013**, *5*, 13146–13152, doi:10.1021/am404180y.
